# Role of Histone Deacetylases in T-Cell Development and Function

**DOI:** 10.3390/ijms23147828

**Published:** 2022-07-15

**Authors:** Monika Pieniawska, Katarzyna Iżykowska

**Affiliations:** Institute of Human Genetics, Polish Academy of Sciences, 60-479 Poznan, Poland; monika.pieniawska@igcz.poznan.pl

**Keywords:** T-cells, histone deacetylases, epigenetics

## Abstract

Histone deacetylases (HDACs) are a group of enzymes called “epigenetic erasers”. They remove the acetyl group from histones changing the condensation state of chromatin, leading to epigenetic modification of gene expression and various downstream effects. Eighteen HDACs have been identified and grouped into four classes. The role of HDACs in T-cells has been extensively studied, and it has been proven that many of them are important players in T-cell development and function. In this review, we present the current state of knowledge on the role of HDACs in the early stages of T-cell development but also in the functioning of mature lymphocytes on the periphery, including activation, cytokine production, and metabolism regulation.

## 1. Introduction

### 1.1. Chromatin and Post-Translational Modifications of Histones

Chromatin, the structure of DNA and higher-order proteins, is involved in the regulation of gene transcription [1]. The structural unit of chromatin is the nucleosome, consisting of a 146 bp DNA segment wrapped around a core histone octamer [2]. Chromatin can take on different structural conformations depending on the epigenetic modifications occurring in both histone tails and globular domains in the nucleosomes [3,4]. Post-translational modifications of histones including acetylation, methylation or phosphorylation modulate chromatin structure and act as marks for recruitment of non-histone proteins to chromatin, leading to either activation or repression of gene expression [5].

Post-transcriptional modifications of histones can modulate chromatin state and gene expression directly or indirectly. Acetylation is a process of adding acetyl groups to histone tails by histone acetyltransferases (HATs) [6]. This process reduces the positive charge on histone tails, resulting in a less dense chromatin structure and thereby weakening the interactions with negatively charged DNA [7]. The hyperacetylated chromatin has a less compact structure and shows the features of transcriptional activity, while the hypoacetylated chromatin is condensed and transcriptionally inactive [8]. The acetylation is correlated with the action of two opposite groups of enzymes: histone acetyltransferases (HATs) and histone deacetylases (HDACs) [9]. Histone methylation is a modification, mainly on the side chains of arginine and lysine. Methyl groups are added to histones by histone methyltransferases (HMT) and reversely removed by histone demethylases (HDMs), and this mediates the transcriptional silencing at heterochromatin sites and affects regulated transcription at euchromatic loci [10,11]. The methylation leads to changes in DNA expression by recruiting the regulatory proteins [12]. This process has been described as influencing many biological processes such as the cell cycle, DNA repair, stress response, and transcription [13]. Histone phosphorylation is a modification of adding phosphate groups, regulated by protein kinases, that can be removed by phosphatases [14]. This process takes place on tyrosine, serine, and threonine, and can be considered an intermediate step in chromosome condensation during cell division or transcriptional regulation [14,15]. This modification establishes interactions between other histone modifications and serves as a platform for effector proteins, which leads to a downstream cascade of events [14,16]. All these modifications are described by epigenetics, a field of science concerning the heritable phenotypic changes without sequence changes in DNA [17]. As epigenetic research progressed, key epigenetic modulators were discovered and divided into three groups: writers-enzymes that add the modification to nucleotide base and specific amino acid residues on histones; erasers-enzymes that remove these marks; and readers-proteins that possess unique domains capable of recognizing specific epigenetic marks in a locus. These enzymes and protein domains are together referred to as “epigenetic tools” [18].

### 1.2. Histone Deacetylases (HDACs)

Histone deacetylases (HDACs) are a group of proteins that remove acetyl groups from histones and allow them to wrap the DNA tightly [19]. HDACs are involved in many important processes such as development and maintaining stable cellular conditions [19]. Moreover, their role in pathological conditions such as genetic diseases and cancer was demonstrated [20]. Histone tails are positively charged due to the amine groups and therfore interact with the negatively charged DNA [21]. The acetylation process neutralizes the charges on histones and decreases the ability to bind to the DNA [11]. Histone deacetylases remove acetyl groups and increase the positive charge on histone tails, promoting the binding between the DNA and histones, which affects the chromatin condensations and repression of the transcription processes [22]. The correlation between HDAC activity and cellular pathways was firstly described in 1997. It was discovered that the overexpression of an HDAC1 in transgenic mouse T-cells led to cell cycle disorders. In this study, HDAC1 overexpression in stably transfected 3T3 cells caused a severe delay during the G2/M phases of the cell cycle [23].

HDACs are classified into four main groups based on their homology to yeast proteins, function, and DNA sequence similarity [24]. Class I includes HDAC1, HDAC2, HDAC3, and HDAC8. The HDAC4, HDAC5, HDAC7, HDAC9, HDAC6, and HDAC10 belong to class II. Class III stands out significantly from other HDACs and is known as the class of Sirtuins, which are homologous to yeast Sir 2 protein and require NAD+ as a coenzyme for activity [25]. Class IV consists of only one enzyme, HDAC11. All HDACs mentioned above, except group III, require a Zn molecule in their active site [25].

### 1.3. T-Cells Maturation and Differentiation

T-cells are white blood cells of the immune system that play a crucial role in the adaptive immune response [26]. They originate from the bone marrow and differ from other lymphocytes by the presence of a T-cell receptor (TCR) on their cell surface [27]. The process of development and maturation of T-cells begins with the hematopoietic stem cells (HSC) in the fetal liver and later in the bone marrow, where HSC can differentiate into multipotent progenitors [28]. T-cells develop through multiple developmental steps in the thymus [29].

All lymphocytes derive from common lymphoid progenitors (CLPs) [30]. The very important step in T-cell maturation is generating the TCR. Each mature T-cell contains a unique TCR that reacts to a random pattern, allowing the immune system to recognize many various pathogens [31]. The TCR consists of two major components: the α-alpha and β-beta chains [32]. The T-cell progenitors in the thymus form a subset of double-negative (DN) cells, lacking both CD4 and CD8 expression, and are referred to as Early T-lineage Progenitors (ETP) [33]. These cells undergo successive development stages characterized by the expression of specific CD4 and CD8 surface markers, starting from the immature double negative (DN) CD4-CD8- thymocytes, through CD4+CD8+ double-positive (DP) thymocytes, and up to mature single-positive (SP) CD4+ and SP CD8+ thymocytes. DN T-cells can be further divided into four different stages (DN1-4) according to the maturation step and expression of CD44 and CD25 on the cell surface with the following immunophenotypes: DN1 (CD44+CD25−), DN2 (CD44+CD25+), DN3 (CD44−CD25+), and DN4 (CD44−CD25−) [34]. The last step of T-cell development is the alternative between turning into a CD4−CD8+ SP T-cell (future CD8 cytotoxic T-cell), a CD4+CD8− SP T-cell (future conventional CD4 T helper cell (Tconv), or a Foxp3+ T-cell (Treg cell) [35].

T-cells are divided into subsets according to their function and expression of certain surface antigens. Helper T-cells (Th cells) expressing CD4 antigen are components of the adaptive immune system [36]. They play a role as “helpers” for other immune cells to release cytokines, which act as mediators to target cells [37]. Their function includes promoting activation of the B lymphocytes to secrete antibodies and activate cytotoxic and memory CD8+ T-cells [38]. Th cells are mainly divided into five subsets, well described by Zhu et al. [39]: Th1 (interferon (IFN)-γ and T-bet), Th2 (interleukin (IL)-4/IL-5/IL-13 and GATA3), Th17 (IL-17/IL-22 and RORγt), Tfh (IL-21 and Bcl6), and Treg (IL-10/transforming growth factor (TGF)-β/IL-35 and Foxp3) [39]. Other Th cells, for example, Th3 and Th22, are also described, but there are some issues in classifying them as lineage subsets [39].

Cytotoxic T-cells (TC, CTL) expressing CD8 antigen are immune components that fight with most intracellular pathogens and are cytotoxic to tumor cells. TC can recognize peptides of the microorganism made within an infected cell when these viral peptides are presented on the cell surface. They destroy their targets directly by inducing them to undergo apoptosis [40,41]. The differentiation of CD8+ cells into subsets also includes cell types other than CTL, for example, T effector memory cells (TEM), T effector cells (TEFF), T central memory cell (TCM), and stem cell memory cells (TSCM) [42].

Regulatory T-cells (Tregs) are a specialized population of T-cells that suppress the immunological response and maintain stable cellular conditions and self-tolerance [43]. It had been indicated that Tregs can inhibit T-cell proliferation, stop cytokine production, and take place in avoiding autoimmune responses, limiting chronic inflammatory diseases [44,45]. Various subsets of Tregs had so far been described, including CD8+ Tregs, natural Tregs (nTregs), Tr1 regulatory cells, and natural killer-like T (NKT) cells. Additionally, they are divided into separate subsets based on their place of origin, for example, nTregs developing in the thymus and iTregs in the periphery from naïve T-cells [46].

## 2. HDACs in T-Cells

### 2.1. Class I HDACs

**Figure 1:**
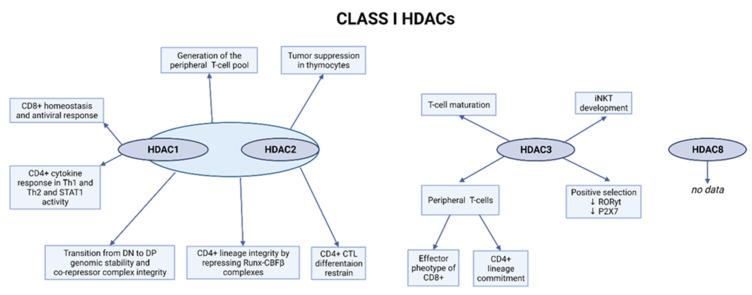
Role of class I HDACs in T-cells. Created with BioRender.

Class I HDACs—HDAC1, HDAC2, HDAC3, and HDAC8—are composed of conserved deacetylase domains, with strong deacetylase activity toward histone proteins [47]. They are ubiquitously expressed and predominantly located in the nucleus. Mostly, they form large co-repressor complexes with other proteins. HDAC1 and HDAC2 are a part of the NuRD chromatin remodeling complex together with ATP-dependent remodeling enzymes CHD3/4, histone chaperones RbAp46/48, CpG-binding proteins MBD2/3, the GATAD2a (p66a) and/or GATAD2b (p66b), and specific DNA-binding proteins MTA1/2/3 [48]. Moreover, they are components of Sin3A co-repressor complex [49], CoREST [50], and the mitotic deacetylase complex (MiDAC) [51], while HDAC3 is recruited to the SMRT/NCoR corepressor complex [52]. Only HDAC8 functions alone without forming a large complex, and its biological role appears distinct from the other class I HDACs [53]. No data on the role of HDAC8 in T-cell development were published, while the role of HDAC1, HDAC2, and HDAC3 in the T-cell maturation and differentiation was widely studied using knockout mice models, and the results confirmed the significance of their role in T-cells (Figure 1).

#### 2.1.1. Role of HDAC1 and HDAC2 in the Early Stages of T-Cell Development

The role of HDAC1 in T-cell development and function was studied using conditional deletion of HDAC1 in the T-cell lineage using Cd4-Cre delete strains [54]. The data presented by Grausenburger et al. revealed that the loss of HDAC1 expression during late stages of thymocyte development and in peripheral T-cells did not lead to alterations in peripheral CD4+ and CD8+ T-cell distribution and numbers, and no difference was detected in the distribution of naïve, effector/memory CD4+ T-cell subsets, or in regulatory T-cells. However, the following study showed that HDAC1 is more essential in early T-cell development [55], as Hdac1f/fLckCre mice used by Tschimarov et al. had reduced numbers of total thymocytes as well as peripheral T-cells.

The role of HDAC1 in both CD8+ and CD4+ T-cells was indicated [54,55]. Tschimarov et al. showed that loss of HDAC1 especially affects the CD8+ and the antiviral response [55]. The population of immature CD8 single-positive thymocytes was increased among Hdac1f/fLckCre thymocytes. CD44hi effector CD8+ T-cells were enhanced in Hdac1f/fCd4Cre mice, yet the INFγ production in this population was not affected. On the contrary, in the total population of HDAC1-null CD8+, IFNγ production was slightly enhanced, but the proliferation rate upon activation, as well as levels of IL2 and TNFα, were similar compared to wild-type HDAC1 CD8+. In vitro differentiation of CD8 effector T-cells was not affected significantly. There was no significant difference in the expression of Runx3, T-bet, and Eomesodermin, key transcriptional regulators of CD8+ differentiation and function, while the upregulation of Perforin did not correlate with increased cytotoxic T-cell activity.

Data by Grausenburger et al. [54] indicated the role of HDAC1 in CD4+ T-cells. HDAC1 activity was shown to be essential for the regulation of the cytokine response in Th1 and Th2 effector cells. Multiple HDAC1 recruitment sites at the Il-4 gene locus and the surrounding cytokine genes in CD4+ T-cells were identified, suggesting a direct control of the IL-4 gene locus by HDAC1 and its possible role in the severity of immune-mediated diseases. To further study the effect of HDAC1 in CD4+ T-cells, Goschl et al. [56] performed the HDAC1 knockout in CD4+ T-cells in mice. The mice were completely resistant to autoimmune disorder EAE (experimental autoimmune encephalomyelitis), even when CD25+ Treg cells were depleted. However, it was not linked to impaired generation of Th17 cells, as it was shown that WT and HDAC1-cKO CD4+ T-cells differentiated into Th17 with similar efficiency. Similarly, the very recent study by Goshl et al. [57] showed that conditional deletion of HDAC1 in the T-cell lineage leads to complete protection of CIA (collagen-induced arthritis), a murine model which resembles rheumatoid arthritis. Reduced serum levels of IL-6 and IL-17 were detected during different phases of the disease, revealing a potential role of HDAC1 in the production of pro-inflammatory cytokines. Upregulation of the chemokine receptor CCR6, which is important for the induction of CIA, was impaired in IL-6 cultured HDAC1-deficient CD4+ T-cells as well as murine and human Th17 cells treated with selective class I HDACi. Another interesting observation made by Goshl et al. in HDAC1-cKO CD4+ T-cells was that they responded differently to anti-CD3 and anti-CD28 TCR stimulation in comparison to WT CD4+ T-cells [56]. The cell proliferation and IL2 production were similar; however, the INFγ was upregulated accompanied by the upregulation of a transcription factor T-bet. Interestingly, RNAseq data showed strong STAT1 upregulation and HDAC1 was detected to be a key player in STAT1 activity in CD4+ T-cells. Not only was the different expression of STAT detected in T-cells of HDAC1-KO mice, but additionally, the level of phosphorylated STAT1 was elevated. What is more, reduced expression of CCR4 and CCR6 was detected, suggesting the negative regulatory role for STAT1 in the regulation of those chemokine receptors.

Not only HDAC1 but also HDAC2 was shown to be essential for T-cell development. Double knock-out of HDAC1/2 resulted in a block in the progression of double-negative (DN) to double-positive thymocytes [58]. Different mechanisms were responsible, including impaired global histone acetylation status and chromosomal stability, and disruption of the cell cycle because of defects in gene regulation and mitosis. The co-repressor complex integrity was also crucial, as loss of not only HDAC1 and HDAC2, but also other central components of Sin3A and NuRD complexes might have disturbed thymopoiesis. Additionally, HDAC1/HDAC2 deletion reduced deacetylase activity, and this correlated with the accumulation of immature CD4low/CD8high and DP cells that failed to undergo positive selection, mainly due to disrupted CD4 gene expression. Similar observations were done by Boucheron et al. [59]. They showed that HDAC1 and HDAC2 were essential to maintaining CD4 lineage integrity by repressing CD8 lineage genes in CD4+ T-cells. Loss of HDAC1 and HDAC2 led to the appearance of MHC class II-selected CD4+ helper T-cells that spontaneously expressed CD8 lineage genes. It was shown that Runx-CBFβ complexes-dependent CD8 effector program was up-regulated in HDAC1-2 cKO CD4 lineage, suggesting that HDAC1 and HDAC2 repress a Runx/CBFβ-dependent CD8 effector program in CD4+ T-cells and thus control the integrity of CD4 lineage T-cells. Moreover, deletion of HDAC1 and HDAC2 led to reduced numbers of peripheral T-cells and to a strong induction of apoptosis in CD4+ T-cells, indicating that HDAC1 and HDAC2 are essential for the generation of the peripheral T-cell pool and the survival of proliferating CD4+ T-cells.

In the early stages of T-cell development, HDAC1 and HDAC2 were also considered to act as tumor suppression [60], as either deletion of the first one or monoallelic loss of the second one in the absence of HDAC1 resulted in spontaneous lymphomagenesis. The authors presented a dosage-dependent model of HDAC1 and HDAC2 in tumor suppression, with the HDAC1 being the key histone deacetylase in thymocytes. Interestingly, although HDAC1 and HDAC2 suppress lymphomagenesis in a dosage-dependent manner, complete inactivation of HDAC1 and HDAC2 abrogates lymphomagenesis as some level of HDAC activity is required for cancer cell vulnerability. Upon HDAC1/2 deletion, Myc was upregulated, and the HDAC1 and HDAC2 were shown to prevent the oncogenic transformation of Myc-overexpressing thymocytes through transcriptional regulation of p53 suppressors.

A very recent study showed that HDAC1 and HDAC2 are key regulators of CD4+ CTL differentiation [61]. Deletions of both HDAC1 and one HDAC2 allele in CD4+ T-cells induced a T helper cytotoxic program that was controlled by IFN-γ–JAK1/2–STAT1 signaling. CD4+ T-cells with HDAC1cKO-HDAC2HET acquired cytolytic activity and displayed enrichment of gene signatures characteristic of effector CD8+ T-cells and human CD4+ CTLs. A stronger induction of CD4+ CTL features was observed within in vivo, murine cytomegalovirus–infection. Finally, using short-chain fatty acids acting as HDAC inhibitors upregulated CTL genes.

HDAC1 and 2 regulate T-cell development, but how are they regulated in T-cells? Several mechanisms were described, and HDAC1 activity in T-cells was shown to be regulated by Foxp3 [62]. Foxp3 is a key transcription factor in Treg development and function. The activity of HDAC1 is reduced by Foxp3, probably by altering its association with co-repressor complexes. HDAC1 was also shown to be directly phosphorylated by Nemo-like kinase (NLK), an evolutionary conserved serine/threonine kinase and a negative regulator of the Wnt signaling pathway that plays a role in T-cell development [63]. Deletion of NLK reduced the number of single-positive (SP) CD8+ thymocytes without any defects in the SP CD4+ thymocyte population. In the CD4+ T-cell population, another mechanism was described, namely, the activation of HDAC1 expression by overexpression of IL-15 [64]. This further led to positive regulation of oncomir miR-21 expression and might have contributed to the malignant transformation of a normal T-cell.

#### 2.1.2. HDAC3 Is Involved in the Positive Selection Process and the Function of Peripheral T-Cells

Many studies showed that HDAC3 is also required for T-cell development. Hsu et al. [65] showed that CD4-cre HDAC3 cKO in mice led to a severe defect in peripheral T-cell numbers, but no effect on intrathymic migration, thymic egress, T-cell survival, or homeostasis was observed. The proportions and absolute numbers of both CD4 and CD8 peripheral T-cells were decreased approximately 10-fold and 6-fold, respectively. Moreover, most HDAC3-deficient naïve T-cells were recent thymic emigrants (RTEs) whose significantly lower maturation marker CD55 expression indicated a block in T-cell maturation [65]. HDAC3-deficient peripheral T-cells also had lower production of TNFα upon stimulation through TCR and CD28, and were targeted for elimination by the classical complement pathway due to a decrease in the sialic acid modifications on the cell surface. Similar T-cell maturation defects were detected in CD4-cre NKAP cKO, indicating that NKAP and HDAC3 work together to regulate T-cell maturation [66]. NKAP is a regulator of gene expression with the C-terminal domain that is associated with HDAC3, and this association is critical for T-cell maturation and iNKT cell development. Defects in thymic T-cell or Treg development were observed in CD4-cre NKAP cKO mice, and CD4-cre driven substitution of endogenous NKAP with NKAP(Y352A) suggests that the ability to interact with HDAC3 is crucial for the function of NKAP. Moreover, both NKAP and HDAC3 were shown to be critical to preventing lipid peroxidation in naïve T-cells and ferroptosis. The involvement of HDAC3 in iNKT cell development was also previously detected [67]. Thapa et al. showed HDAC3 to be involved in the autophagy required for the proper development of iNKT cells. iNKT cells differentiate into effector subsets NKT1, NKT2, and NKT17 in the thymus. Loss of HDAC3 leads to a decreased autophagy and a severe defect in NKT1 effector cells, while NKT2 and NKT17 have decreased ability to produce IL-4 and IL-17, respectively.

It was shown that HDAC3 is especially required for a positive selection process [68]. HDAC3–cKO mice showed an increased frequency of DN cells, a decreased frequency of CD4SP thymocytes, and immature CD8SP thymocytes. The study indicated that HDAC3 down-regulates RORγt because of histone deacetylation in the promoter region, which is a crucial step in positive selection. In the HDAC3-deficient mice, RORγt was not down-regulated upon TCR stimulation at the DP stage, probably due to hyperacetylation present in the promoter region. Similar results were obtained by Stengel et al. [69]. Impaired maturation of DN cells, an increase in immature SP CD8+ cells, and impaired maturation of DP cells, causing a dramatic decrease in SP CD4+ and CD8+ cells, were observed in HDAC3 KO mice. Authors analyzed global gene expression in DP thymocytes and detected deregulation of many genes required for positive selection, T-cell function, and cell cycle progression. Results also suggested that impaired TCR signaling as transgenic mouse expression of a combined TCRαβ transgene provided a high level of complementation of thymocyte development. Another study revealed that HDAC3 promotes DP survival by suppressing P2X7 receptor expression in DP thymocytes. HDAC3 and RORγt regulate the expression of the P2rx7 gene by interaction with its enhancer [70]. HDAC3-deficient DP thymocytes had increased acetylation in the P2X7 gene locus and increased expression of the purinergic receptor P2X7. Those cells were more sensitive to high concentrations of extracellular ATP and P2X7 receptor-induced cell death. Interestingly, in HDAC3-deficient DP thymocytes, RORγt, which is upregulated in the absence of HDAC3, was bound to the P2rx7 enhancer and promoted P2X7 receptor expression.

It is worth mentioning that HDAC3 is required not only for positive selection but also for the whole NCOR1 corepressor complex [71]. NCOR1 KO mice also showed impaired positive selection; however, the mechanism was different, in that the RORγt was properly downregulated in NCOR1-cKOCd4 thymocytes. Similarly, peripheral T-cell numbers were reduced in NCOR1-cKOCd4 mice; however, CD55 expression levels were normal on peripheral NCOR1-cKOCd4 T-cells, indicating that NCOR1, unlike HDAC3, is dispensable for post-thymic T-cell maturation.

HDAC3 was shown to be required not only for proper T-cell development but also for peripheral T-cells. Tay et al. [72] described the role of HDAC3 in regulating the effector phenotype of CD8 T-cells after activation. CD8 T-cell effector was previously shown to be regulated by epigenetic modifiers. In this study, HDAC3 inhibition was performed, and the results showed that HDAC3 is a negative regulator of CD8+ T-cell cytotoxicity. Increases in the cytotoxicity-associated functional markers granzyme B, IFN-γ, and CD25 were detected upon using an HDAC3-specific inhibitor. This inhibition of CD8 T-cell cytotoxicity by HDAC3 was confirmed by different experimental approaches, and further experiments confirmed that HDAC3 inhibits the cytotoxicity program early following activation. The next thing under investigation was whether loss of HDAC3 could alter the dynamics of the CD8 T-cell response, and the analysis showed that HDAC3 is required for CD8 T-cell persistence following the resolution of acute infection [72]. RNA-seq data indicated the role of HDAC3 in the negative regulation of gene programs associated with CD8 T-cell cytotoxic effector function, including direct mediators of cytotoxicity (Gzmb, Gzmc, Prf1) and transcription factor genes that promote an effector phenotype in CD8 T-cells (Prdm1, Id2). Not only the expression was altered but also epigenetic marks; in particular, there was an increase in the global histone mark H3K27ac at several gene-encoding regulators of CD8 T-cell activation, effector function, and differentiation, including transcription factors, regulators of T-cell receptor signaling, and surface receptors. A model was proposed by authors in which HDAC3 epigenetically regulates a network of genes including Runx3 and Prdm1 in CD8 T-cells during CD8 T-cell activation to inhibit differentiation into cytotoxic effector cells.

The function of HDAC3 for CD4+ cells was also confirmed [73], as HDAC3-deficient thymocytes failed to induce the CD4-lineage program and committed to the CD8-lineage. It was shown that HDAC3 binds to regulatory elements of CD8-lineage-promoting genes Runx3 and Patz1 in DP thymocytes. In WT thymocytes, HDAC3 associates with Runx3 and Patz1 in DP thymocytes to restrain CD8-lineage gene expression. After positive selection, HDAC3 stays bound to these regions in CD4SP thymocytes for CD4-lineage commitment, while in CD8SP thymocytes, HDAC3 no longer binds to these regions for CD8-lineage commitment. Deletion of HDAC3 increased histone acetylation at Runx3 and Patz1, and as a result, Runx3 was pre-maturely expressed in DP thymocytes and cells committed to the CD8-lineage.

### 2.2. Class II HDACs

In class II HDACs (Hda1-like), the following proteins are distinguished: HDAC4, HDAC5, HDAC6, HDAC7, HDAC9, and HDAC10. Those enzymes have known deacetylase domains with high homology to Hda1, which is found in many yeast species and is responsible for the deacetylation of lysine residues on the N-terminal part of the core histones [74]. All of them are divided into IIa and IIb subclasses [75]. Subclass IIa, including HDAC4, HDAC5, HDAC7, and HDAC9, has a special domain in the N-terminus which forms a binding site for the DNA-binding transcription factor MEF2 and the subsequent 3~4 phosphorylation sites that act as regulatory signals for the association of 14-3-3 proteins [47]. They can move between the nucleus and cytoplasm as a response to proper regulatory signals [76]. Class IIb HDACs, including HDAC6 and HDAC10, have a unique long extension at the C-terminus; this extension is the so-called tail domain. Both enzymes can be localized in the cytoplasm, and although they belong to the same IIb class, they are a little different. The HDAC6 contains two deacetylase domains and a C-terminal zinc finger ubiquitin-binding domain, while HDAC10 has only one deacetylase domain and a leucine-rich repeat domain at its C-terminus, which is responsible for its cytoplasmatic enrichment [47,77]. HDAC6 can be localized in the nucleus and is involved in regulating the transcription process. However, how HDAC6 shuttles between the nucleus and cytoplasm is still not fully understood, and neither is the modulation of its enzymatic activity [78].

#### 2.2.1. HDAC4 Is Expressed in the Multiple T-Cell Lineages but Is Not a Key Regulator of T-Cell Biology

The role of HDAC4 in T-cell development and function was studied using T-cell-specific HDAC4-deficient mice. Data presented by Liu et al. [79] reported that HDAC4 is expressed in the multiple T-cell lineages, including thymic CD4- CD8- DN and CD4+ CD8+ DP, thymic and splenic CD4+ SP cells, and CD8+ SP T-cells, as well as TCR-β+ Tet+ iNKT cells [79]. The dynamic change of HDAC4 expression during T-cell differentiation was observed, suggesting its role in T-cell development and function [79]. However, it was indicated that Tconv cells can develop properly in absence of HDAC4. The experiment was conducted on generated T-cell-specific HDAC4-knockout (KO) mice in comparison to WT which were designated as HDAC4fl/fl (HDAC4 WT) [79]. HDAC4 expression was lower in thymic CD4+ SP T-cells and iNKT cells in HDAC4 KO mice, but there was no decline in its expression in thymic CD8+ SP T-cells. In addition, there were no significant alterations in the frequencies and numbers of thymic CD4-CD8- DN and CD4+CD8+ DP. The CD4+ and CD8+ T-cell distribution were similar between HDAC4 KO and WT mice [79]. To finally prove that the absence of HDAC4 did not affect conventional T-cell development, corresponding percentages of thymocytes at different development stages based on positive selection were identified between HDAC4 KO and WT mice [79]. Moreover, it was shown that HDAC4 is also not necessary for iNKT cell development, and no significant differences were identified in the percentages and absolute numbers of iNKT within the spleen and thymus between the tested group of HDAC4 KO mice and their WT counterparts [79]. It was also investigated whether the HDAC4 could play a role in Th cell polarization. The in vitro stimulation of splenocytes of KO and WT mice with PMA and ionomycin did not show any significant differences in the production of TNF-α, IFN-γ, IL-4, or IL-17, indicating that HDAC4 is not essential for Tconv cell function and polarization [79].

Data published by Guo et al. [80] suggested the role of HDAC4 in CD4 + T-cells based on hypermethylation of the HDAC4 region in CD4 + T-cells of rheumatoid arthritis patients [80,81]. The DNA methylation profiling indicated that HDAC4 was one of the most hypermethylated genes and its expression could be decreased in the RA patients, but HDAC4 expression was not evaluated in performed research [80,81].

#### 2.2.2. Loss of HDAC5 Weakens the Tregs and Tconv Function

**Figure 2:**
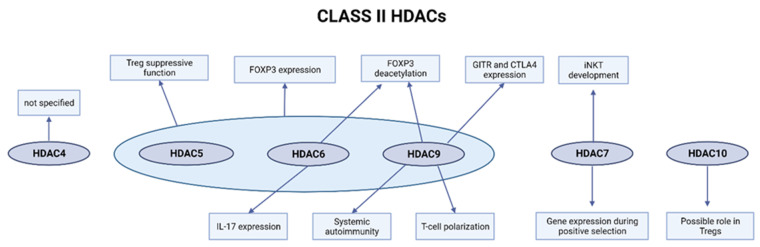
Role of class II HDACs in T-cells. Created with BioRender.

The role of HDAC5 in T-cells function was explored by Xiao et al. [82]. The development and homeostasis of T-cells in HDAC5-deficient mice appeared normal, but their Tregs showed impaired suppressive function both in vivo and in vitro (Figure 2) [82]. HDAC5-deficient mice developed a normal population of CD4+ and CD8+ T-cells, and additionally, the subsets of activated CD4+ and CD8+ T-cells with the CD4+Foxp3+ Treg cells were almost equal to WT mice [82]. Upon isolation of CD4+ CD25+ Tregs, it was observed that they lost their suppressive function in vitro and the weaker HDAC5−/− Treg function was also noted. The authors confirmed diminished HDAC5−/− Treg function in allograft studies, as WT Treg cells were able to prevent rejection of the mismatched cardiac allograft at a 2:1 Tconv to Treg ratio, while the HDAC5-deficient Treg cells were unable to maintain the allografts [82]. In the same paper, HDAC5−/− Tconv did not demonstrate an evident difference in function, including proliferation under CD3ε/CD28 stimulation or cytokine production under PMA/ionomycin stimulation, but a small difference was observed in the case of proliferating T-effector cells, where those lacking HDAC5 were more resistant to Treg-mediated suppression [82]. Despite no difference in functions, the authors indicated that the effect of HDAC5 loss can be noticed in one case. CD4+ Tconv cells without HDAC5 differed significantly from WT controls when exposed to polarizing conditions to promote iTreg development; it was observed that HDAC5 loss weakens the ability of T-effector cells to convert into iTreg cells, but without affecting Th17 conversion [82]. Moreover, it was also found that HDAC5 loss led to a Foxp3 decrease in Tregs [82]. HDAC5−/− Tregs showed no significant differences in Foxp3 or cytotoxic T-lymphocyte-associated protein (CTLA)-4 gene expression, although the authors noticed a trend towards lower Foxp3 mRNA expression [82]. Experiments on the protein level indicated Foxp3 protein reduction in HDAC5−/− Tregs and also slightly higher phosphorylation of Foxo1 protein [82]. The authors also showed that the lack of HDAC5 disturbed the ability of CD8+ T-cells to produce IFN-γ in response to CD3ε/CD28 or PMA/ionomycin stimulation [82].

#### 2.2.3. HDAC6 Is Involved in Treg Suppressive Function and FOXP3 Expression and Deacetylation

The role of HDAC6 in T-cell function, development, and homeostasis was described in several publications. Beier et al. [83] indicated that the heat shock response mediated by HDAC6 inhibition improved Treg suppressive function. The deletion of HDAC6 and also HDAC9 led to hyperacetylation of Hsp90 at Lys294 in Tregs and also the nuclear translocation of HSF-1. The authors also hypothesized that the HDAC6 might interrupt the Treg biology independently of the heat shock response [83]. It was detected that HDAC6 was translocated from cytoplasm to the nucleus of Tregs, which were activated through CD3ε and the coreceptor CD28 [83]. This observation suggested that HDAC6 might have a nuclear function by targeting acetylation of nuclear proteins important to Treg’s function [83]. It was detected that Foxp3 protein was higher in HDAC6−/− Tregs compared to wild-type controls and that the level of acetylated Foxp3 was higher in the absence of HDAC6 [83]. This suggested that HDAC6 deacetylates Foxp3 and that its loss promotes the Foxp3 acetylation, with an increase in the resistance of Foxp3 to proteasomal degradation [83]. Based on these observations, HDAC6 could be involved in the regulation of additional transcription factors which are necessary for Tregs as promotion of the nuclear translocation of phosphorylated cAMP response element-binding protein (CREB) [83].

Data published by Zoeten et al. [84] indicated that HDAC6 genetic targeting did not affect Treg number or significantly modify Foxp3 expression, but it affected the phenotype of Tregs and enhanced Treg suppressive function in vitro. The effect of targeting HDAC6 was also investigated in vivo using HDAC6 inhibitors and HDAC6−/− cells, but it was observed that it did not weaken CD4+ T-cell migration, proliferation, or conversion into Foxp3+ Tregs of transferred cells [84]. Additionally, the study revealed that targeting HDAC6 could affect the development and progression of murine colitis (MC), a disease strongly correlated with FOXP3. It was shown that targeting HDAC6 could prevent the development of MC and that the treatment using HSP90i or HDAC6i could rescue mice that had already developed the disease. The authors also investigated the effects of HDAC6 targeting on major histocompatibility complex (MHC) mismatched transplant models; they used a cardiac allograft model in which acute rejection in mice was mediated by adoptively transferred B6 T-cells but could be suppressed by co-transfer of B6 Tregs [84]. It was discovered that the acute rejection or long-term engraftment of cardiac allografts could be determined by the presence or absence of HDAC6 within the adoptively transferred Treg population, and HDAC6 targeting could result in long-term allograft survival in MHC-disparate combinations [84,85].

The role of HDAC6 in the interleukin expression was confirmed by Yan et al. [86]. They described that even though the T-cells function and the development of mice with HDAC6 deletion or inhibition were normal, the expression of IL-17 in γδ T-cells was promoted [86]. The authors investigated the role of HDAC6 in the development of T-cells by analyzing the populations of CD4+ and CD8+ from lymphoid and non-lymphoid tissues. The results showed that loss of HDAC6 did not significantly alter the distribution of the CD4+ or CD8+ T lymphocytes [86]. They compared the IL-17 and interferon-γ (IFN-γ)-producing subpopulations of γδ T and CD4+ T-cells using flow cytometry [86]. The number of IL-17-producing CD4+ T-cells in the spleen was only slightly increased in HDAC6 KO mice, but the number of IL-17-producing γδ T-cells was significantly higher [86]. On the other hand, the lack of HDAC6 did not alter the number of IFN-γ-producing γδ T and CD4+ T-cells. These data suggested that HDAC6 could play a crucial role in the regulation of IL-17 production and expression [85,86].

#### 2.2.4. HDAC7 Regulates Gene Expression during Positive Selection and iNKT Development

Several studies confirmed that HDAC7 is also required for T-cell development. Dequiedt et al. published that HDAC7 is highly expressed in CD4+CD8+ DP thymocytes and inhibits Nur77 expression [87]. Kasler et al. [88] showed that HDAC7 is exported from the nucleus during the positive selection of thymocytes, regulating the DP thymocyte survival and gene expression changes associated primarily with the positive selection of TCR signals. The authors investigated the subcellular localization of HDAC7 in thymocytes that were receiving different types of in vivo TCR signals [88]. In WT DP thymocytes, which received no TCR stimulation, HDAC7 was present in the nucleus in almost all cells, while in the CD4 SP thymocytes, HDAC7 localization was cytoplasmic [88]. This discovery suggested that HDAC7 could regulate gene expression during positive selection and that the nuclear localization of HDAC7 could mediate a long-term change in the differentiation of thymocytes [88]. Moreover, the lack of HDAC7 function led to a significant defect in the ability of DP thymocytes to become positively selected and to mature to the SP stage [88]. The authors hypothesized that the deletion of HDAC7 could cause changes in the TCR activation thresholds which affect the positive and negative selection and reduce the population of TCRs that can mediate survival [88]. The effect of the HDAC7 deletion was investigated on lck-cre transgenic mice with three TCR specificities which are positively selected in normal conditions. The introduction of a TCR transgene reduced the negative effect of HDAC7 loss on thymocyte maturation [88]. This observation suggested that the deletion of HDAC7 does not primarily impair positive selection by changing the cell fate mediated by a particular TCR specificity. Another explanation was that the defective TCR generation by HDAC7 KO thymocytes might result from a defect at the β-selection checkpoint. However, in the case of β-selection, HDAC7 thymic deletion with the same lck-cre mice system was not sufficient to bypass this selection process, and any defect in the generation of positively selected TCRs in the HDAC7 KO takes place after β-selection [88]. As a result, it was proven that HDA7 deletion did not affect either the TCR affinity threshold for positive selection or the β-selection process. Further experiments showed that the lack of HDAC7 during the DP stage caused a shortening of the thymocyte lifespan that undermined the thymocytes’ ability to be positively selected and truncated the TCR α-chain repertoire [88,89,90]. It was also indicated that HDAC7 can mediate gene expression associated primarily with positively selecting TCR signals and can regulate DP thymocytes cell survival through many TCR-regulated pathways, and also that the loss of HDAC7 during T-cell development caused an increase in apoptosis causing inefficient positive selection [88,89,90]. For example, altered activity of various effectors of the TCR signaling such as p38 MAPKs caused apoptosis of DP thymocytes, leading to defects in both T-cell numbers and T-cell repertoire [88]. To sum up, HDAC7 appears to be a negative regulator of the coupling between TCR engagement and the downstream signaling cascades that determine cell fate [88].

In two additional papers published by Kasler [89,90], the importance of HDAC7 in T-cells functionality was also described. It was observed that HDAC7 controls the thymic effector programming in Natural Killer T-cells (NKT) and interferes with the process of iNKT development [89]. HDAC7 loss reduced iNKT number in thymocytes and expanded an innate-memory CD8 population compared to wild-type controls [89]. Moreover, it was discovered that HDAC7-ΔP blocked innate effector development in iNKT and lead to conversion into naïve-like T-cells [89]. Authors also indicated that HDAC7 regulated genes in glycolipid-reactive cells, which is relevant to innate effector function, inflammation, and also autoimmune processes [89]. It was indicated that the impaired phosphorylation pathway of HDAC7 made it unable to shuttle into the cytoplasm during T-cell development, and as a result, the negative selection process was disrupted which could lead to autoimmunity [89,90]. The authors investigated the influence of blocking TCR-dependent nuclear export during the thymic selection, based on the expression of HDAC7-ΔP in the thymocytes, and it was observed that in this case, thymocytes manifested a blocking in thymic selection but still could undergo positive selection with permit escape of autoreactive T-cells to the periphery [90]. It was also indicated that HDAC7-ΔP affected gene expression changes correlated with both positive and negative selection, and blocked MAP kinase activation after strong TCR engagement [90]. Experiments performed in vivo on mice showed that HDAC7-ΔP TG mice developed lethal multi-organ autoimmunity, manifesting with lethal exocrine pancreatitis and visceral auto-aggression [90].

The role of HDAC7 in T-cells’ function was also studied by Myers et al. [91]. It was discovered that tonic signals through LAT exported the HDAC7 from the nucleus of CD4+ T-cells and that it was phosphorylated in CD4+ T-cells [91]. HDAC7 repressed INFγ, and ex vivo stimulation for cytokines revealed that insufficiency of HDAC7 led to an increase in percentages of CD4+ T-cells producing IFN-γ [91]. Microarray gene expression in LATWT, LATNEG, and LATY136F mice showed no significant differences in HDAC7 mRNA levels, just as the level of HDAC7 between in vitro generated Th2 as compared with Th1, Th2, and Th17, and Treg or Th0 also did not manifest significant changes [91]. On the other hand, it was indicated that tonic regulation of HDAC7 influenced both Nur77 expression and CD4+ proliferation, tonic LAT-HDAC7 maintained Irf4 in naïve T-cells, and this regulation limited Th2 polarization of CD4+ cells [91].

#### 2.2.5. Role of HDAC9 in Treg Function, T-Cell Polarization, and Systemic Autoimmunity

Many studies investigated the role of HDAC9 in the context of T-cells’ function or development. Tao et al. [92] indicated Tregs’ suppressive activity using in vivo conditions. Higher expression of FOXP3, GITR, and CTLA4, and increased FOXP3 acetylation were detected comparing Hdac9−/− mice Tregs to WT controls. Upon using HDAC inhibitors (HDACi), the proportion of Foxp3+ CD4+ T-cell numbers increased. Treg cells had significantly higher overall HDAC activity than CD4+ CD25– T-cells, and the HDAC activity of both was blocked by trichostatin A (TSA) [92]. Expression of all HDACs was measured in Treg cells, and significant differences were detected in the case of HDAC9. mRNA level encoding HDAC9 was higher in Tregs than in non-Treg cells. TCR stimulation caused a 90% decrease in HDAC9 mRNA expression in non-Treg cells, but in Treg cells, it was higher [92]. The data also showed that upon Treg activation, nuclear export of HDAC9 protein was induced. HDAC9 was localized in the nuclei of resting Treg cells but shuttled to the cytoplasm after TCR activation [92]. Moreover, the HDAC9 role in the regulation of Treg function was investigated using Hdac9−/− mouse model. The proportion of CD4+ Foxp3+ T-cells in lymphoid tissues of Hdac9−/− mice was increased by almost 50% compared to WT control. Additionally, Hdac9−/− Tregs were more suppressive than controls [92].

Data presented by Yan et al. [93], who investigated the role of HDAC9 in systemic autoimmunity, showed that it could cause a cascade of several actions, including Th1 cell-type cytokine decrease, increased Th2 cell-type cytokine production, and reduced T-cell activation. The real-time PCR analysis performed on isolated CD4+ T-cells and splenocytes showed that loss of HDAC9 decreased mRNA levels of IFN-gamma and IL-12 and increased expression of IL-4 and GATA3 [93]. To investigate the role of HDAC9 in T-cell polarization, CD4+ T-cells from KO and WT mice were cultured in neutral and Th1- or Th2-polarizing media. HDAC9-deficient CD4+ T-cells produced more IL-4 mRNA and IL-4-producing Th2 cells under Th2-polarizing conditions, but there was no significant change in the Th1 cells in Th1- polarizing conditions [93]. To determine the role of HDAC9 in systemic autoimmunity, authors measured the expression of HDAC9 in splenocytes from MRL/lpr mice and control MRL/MpJ mice, and generated MRL/lpr mice lacking the HDAC9 gene [93]. It was noticed that HDAC9 was overexpressed in various subsets of CD4+ T-cells in MRL/lpr mice and human lupus samples compared to healthy controls [93]. HDAC9 expression was also measured between disease progression in several organs, such as the spleen or the kidney, and purified subsets of CD4+T-cells from different age groups of MRL/lpr mice; it was observed that HDAC9 expression was increased in the kidneys and spleens of those mice [93]. Moreover, the authors indicated that HDAC9 deficiency increased hyperacetylation at specific histone residues, i.e., H3K9, H3K14, and H3K18 of the histone H3. The lysine residues of investigated HDAC9 KO and MRL/lprHDAC9+/+ mice appeared to be hyperacetylated in the mice lacking HDAC9 [93]. The specificity of those changes between various organs was also indicated, revealing the hyperacetylation in splenocytes and kidneys [93]. The study also indicated that the loss of HDAC9 decreased activated plasma cells and T-cells, and overall improved the serological and clinical autoimmune phenotype of HDAC9 KO mice, influencing the modification of genes that are involved in follicular and extrafollicular CD4+ T-eff cells [93]. HDAC9 was also described as down-regulating inducible T-cell co-stimulator (ICOS) and its loss decreased expression of chemokines and cytokines by up-regulating PPAR-γ [93].

Zoeten et al. [94] indicated that HDAC9 did not affect T-cell development and cell cycle but increased suppressive activity that correlated with FOXP3 and IL-10 expression in Hdac9–/– Tregs [94]. The data published by Beier et al. [83] indicated that HDAC9 loss stabilized and promoted STAT5 acetylation and phosphorylation in HDAC9−/− Tregs, and also its transcriptional activity [83]. Beier et al. [95] proved that deletion of HDAC9 in Treg cells increased gene expression in oxidative phosphorylation (OXPHOS) and displayed increased cellular respiration in Tregs (mitochondrial respiratory function of Tregs), but the same effect was not seen in conventional T-cells (Tconv) [95].

#### 2.2.6. Role of HDAC10 in Treg Functionality and Immunosuppression

Not many data on the role of HDAC10 in T-cell development were published. Dahiya et al. [96] investigated its role in Tconv cells and Treg cells in the case of autoimmune colitis. It was shown that HDAC10 deletion neither affected Tconv cells nor Tregs metabolism but improved the Tregs functionality. Mice with HDAC10 loss were observed to have an equal proliferation rate of CD4+ and CD8+ compared to WT control [96]. Moreover, there was also no difference in the production of IL-2 and IL-gamma [96]. In comparison, between HDAC10−/− mice and WT, loss of HDAC10 in Tregs led to stronger suppressive function against effector T-cells [96]. It was indicated that HDAC10 co-precipitates with Foxp3 in the 293T cell line, although the authors could not confirm an increase in Foxp3 protein acetylation [96]. Moreover, it was noticed that Treg cells with a lack of HDAC10 alleviated the colitis using in vivo models, which could indicate that HDAC10 deletion possibly had an immunosuppressive effect [96].

### 2.3. Class III HDACs

HDACs class III, Sirtuins, are proteins with homology to yeast Sir 2 that require NAD+ as a coenzyme for their activity [25]. So far, seven Sir2-like proteins have been discovered in humans and described as SIRT1-SIRT7 [25]. Sirtuins play an important role, both direct and indirect, in transcriptional regulation [97]. They are involved in multiple processes, including apoptosis, stress tolerance, hormone responses, differentiation, and development [97], and have been detected to regulate T-cells’ function and development [98].

#### 2.3.1. SIRT1 Is a Negative Regulator of T-Cell Activation

**Figure 3:**
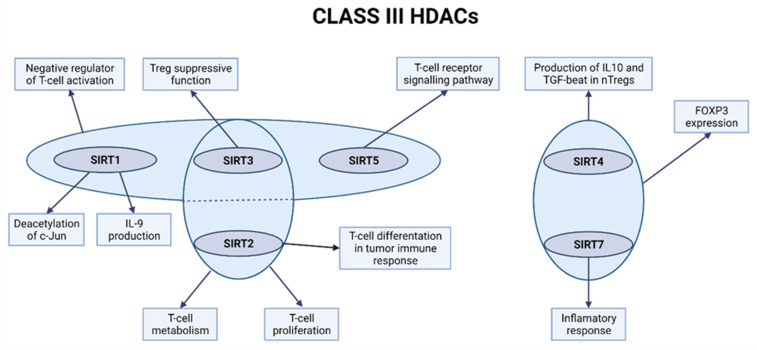
The role of class III HDACs in T-cells. Created with BioRender.

Sirtuin1 is considered to influence T-cell activation, tolerance, and inhibition of transcriptional activity (Figure 3) [99]. Data presented by Kong et al. showed that mice lacking Sirt1 present a pro-inflammatory T-cell phenotype with increased proliferation [99]. Moreover, in the paper published by Zhang et al., it was proven that Sirt1 negatively regulates T-cell activation in vivo, where Sirt1 deficiency in mice is correlated with an increased number of activated T-cells and breakdown of CD4 + T-cell tolerance. The use of the Sirt1−/− mice model in the study proved that Sirt1 influences the T-cell-dependent immunity with a suppressing effect and that Sirt1 acts as a suppressor of Activator-Protein 1 (AP-1) in T lymphocytes. It was also observed that Sirt1 presented an inhibitory effect on the T-cells activation by limiting the acetylation of c-Jun, which is a transcription factor [98] Wang et al., based on research on Sirt1flox/floxCd4-Cre mice model, indicated that glycolysis dependent on SIRT1 was associated with Th9 differentiation [99]. Moreover, loss of Sirt1 in CD4+ T-cells increased IL-9 production and glycolytic metabolism [98,99].

The inhibition of Sirt1 was also associated with Th9 differentiation and IL-9 production in the case of HIF1-alpha activity, which was described in a paper published by Wang et al. [100]. In addition, Wilhelm et al. [101] reported that SIRT1 lacking CD4+ T-cells produced IL-9, which was involved in inflammation of airways, based on its allergic background [100,101]. Purwar et al. [102] indicated that the IL-9 produced from SIRT-1-deficient CD4+ cells was inhibiting malignant tumor growth in the case of melanoma [100,102]. Data presented by Sequiera et al. [103] showed that mice lacking the Sirt1 gene demonstrated increased proliferation of T-cells and higher IL-2 production [103].

#### 2.3.2. SIRT2 Regulates T-Cell Metabolism and Tumor T-Cell Immune Response

Data published by Hamaidi et al. [104] indicated the function of Sirt2 in the regulation of T-cell metabolism of Tumor-Reactive T-cells. The Sirt2 expression was detected to be upregulated in peripheral blood mononuclear cell samples, during both T-cell maturation and activation [104]. The authors observed that Sirt2−/− T-cells presented an increased proliferation rate following antigenic stimulation in comparison to WT controls. Moreover, Sirt2 interacted with the enzymes of glycolytic pathways in T-cells. The deletion of Sirt2 promoted CD4+ and CD8+ TM cell formation ex vivo, which was associated with cell survival and decreased apoptosis [104,105]. Sirt2 was also investigated by Jiang et al. in the regulation of T-cell differentiation in tumor immune response, indicating that it may participate in the cancerous immune response by regulation of T-cell differentiation [106]. In samples collected from the blood of breast cancer patients, the expression of Sirt2 was lower in comparison to healthy individuals, and Sirt2-deficient mice demonstrated decreased TEM cells with increased naïve T-cell levels [106].

#### 2.3.3. Role of SIRT3 in Promoting T-Cell Responses and Reducing Transplant Rejection

The role of Sirtuin 3 in T-cells was investigated in a study performed by Tubai et al. [107] which indicated that lack of Sirt3 did not have an impact on T-cell differentiation, development, or activation (under resting) in vivo conditions [107]. In comparison to in vitro studies, it was noticed that Sirt3 has a slightly promoting effect on T-cell responses, by reducing proliferation in Sirt3−/− T-cells [107]. The authors also observed that Sirt3−/− mouse model reduced graft-versus-host disease (GVHD) severity in comparison to T-cells from control donor mice, which could suggest the ability of Sirt3 to improve the outcome of the transplant [105]. Additionally, the protective effect of allogeneic Sirt3−/− T-cells was noticed to reduce the T-cell proliferation and CXCR3 expression, which is an early marker of T-cell activation, with no influence on cytokine secretion [105,107]. Beier et al., investigating the role of mitochondrial energy metabolism in Treg cells, indicated that loss of Sirt3 weakens the Treg function in vitro and in vivo, emphasizing its importance in Treg. The Sirt3 itself promotes the Treg suppressive function by induction of oxidative phosphorylation (OXPHOS) metabolism. OXPHOS is important for both T-cell proliferation and the Treg suppressive function [107].

#### 2.3.4. Role of SIRT4 and SIRT6 in Treg Regulations in Case of Traumatic Spinal Cord Injury

Not many data on the role of Sirtuin 4 and Sirtuin 6 in the adaptive immune response have been published. Hamaidi et al., based on the correlation between glutaminolysis in Th17 differentiation and Treg development, concluded in a review that Sirt4 can play a role in inflammatory regulations, mainly expecting a proinflammatory phenotype while lacking the Sirt4 [105]. A recent study published by Lin et al. described the correlation between traumatic spinal cord injury (SCI) and Sirt4 [108]. Based on the Foxp3-GFP mice model, it was indicated that Sirt4, the same as Sirt6, was up-regulated in infiltrating Treg cells after the spine injury, which could suggest that the environment of spinal injury promotes the transcription of both Sirtuins in Tregs [108]. Sirt4 overexpression was also found to reduce the IL-10 and TGF-beta in natural Treg (nTreg) cells [108]. The authors mentioned that nTreg cells could be predominant in the post-SCI infiltrating Treg cells, so for a better understanding of the role of Sirt4 in this action, they investigated the effect of this Sirtuin on induced Treg cells [108]. The results indicated that Sirt4 overexpression decreased the Foxp3 expression level [108].

#### 2.3.5. The Potential Involvement of SIRT5 in the T-Cell Receptor Signaling Pathway and SIRT7 in Inflammation Processes

Not many data on the role of Sirtuin 5 in the immune response were investigated, some studies reported correlations between Sirt5 and T-cells. The paper published by Heinonen et al. [109] summarized that Sirt5 deficiency did not affect the development of the major T-cells in the thymus [109]. In a paper by Wang et al. [110], the proteome data and network analysis indicated that Sirt5 played a role in the T-cell receptor signaling pathway [110]. The authors also determined that a deficiency of Sirt5 induced stronger T-cell activation and played a role in the regulation of the differentiation of CD4+ regulatory Treg cells and Th1 cells [110].

Sirtuin 7 is the least studied member of class III HDACs, especially in the case of its role in modulating adaptive immune response and impact on T-cell development and functions. This Sirtuin is considered to influence the inflammation process [111]. Sánchez-Navarro et al. [112] indicated that in the case of Acute Kidney Injury (AKI), Sirt7 was associated with a reduction of immune cells infiltration, especially with a significant increase in total T-cell infiltration [112]. Data published by Vakhrusheva et al. [113] also supported the thesis that Sirt7 may be involved in the inflammatory response, suggesting its important role in cardiac inflammation [113].

### 2.4. Class IV HDACs

Only one HDAC belongs to class IV, HDAC11, which is the smallest HDAC. It is mainly localized in the nucleus, but it could be also detected in the cytoplasm, for example, in resting CD4+ T-cells [114,115]. Most of the HDAC11 protein sequence encodes the HDAC catalytic domain, while N- and C-terminal extensions do not contain any predicted protein binding sites [116]. It does not interact with typical HDAC co-repressor complexes, but it was shown to interact with HDAC6. HDAC11 was described to be not only the epigenetic repressor but also shown to act as a nonhistone protein deacetylase and long-chain fatty acid deacetylase. It is the only HDAC involved in mRNA processing and splicing through binding to the RNA-binding proteins DICER and the SMN complex [116].

#### HDAC11 Is Involved in T-Cell Activation and Treg Function

**Figure 4:**
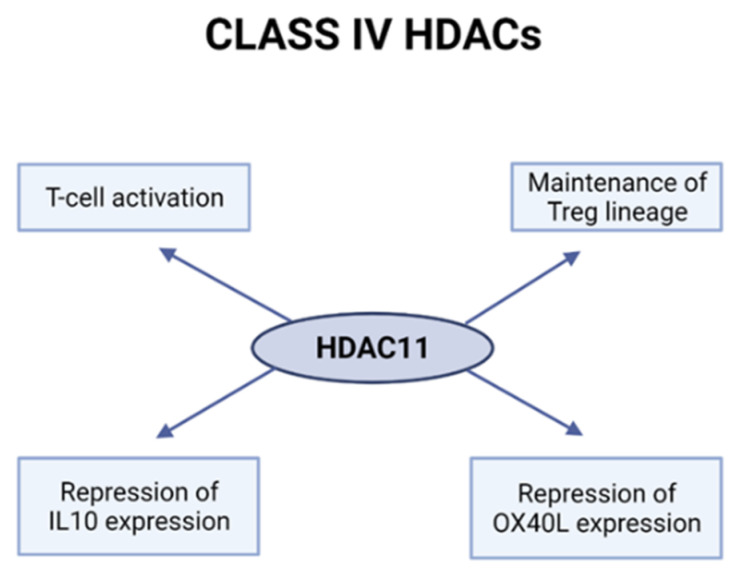
Role of class IV HDACs in T-cells. Created with BioRender.

HDAC11 was shown to indirectly regulate the function of T-cells by being a repressor of IL10 expression in antigen-presenting cells (APC) (Figure 4) [117]. Overexpression of HDAC11 decreased the level of IL-10 and induced inflammatory APCs to prime naïve T-cells and restore the responsiveness of tolerant CD4+ T-cells. On the other hand, disruption of HDAC11 in APCs upregulated IL-10 and impaired the antigen-specific T-cell responses. The same group investigated the role of HDAC11 in T-cells [118]. Two mice models were used in a study: HDAC11-EGFP transgenic and HDAC11 knockout (KO) mice. The analysis using the EGFP-HDAC11 transgenic reporter mouse with EGFP expression driven by the HDAC11 promoter showed that the expression of HDAC11 is decreased in activated and effector T-cells compared to naïve T-cells. In the HDAC11KO mice, the percentage of effector subsets following activation in the T-cell population was increasing. These results suggested the negative regulatory role of HDAC11 in T-cell activation. Moreover, T-cells lacking HDAC11 had increased proliferation and proinflammatory cytokine production. HDAC11 was also proved to be a regulator of T-bet and Eomes; it was shown to be recruited to the promoter regions in resting T-cells but disassociated after T-cell activation. Those genes are known to be involved in GVHD, so the enhanced alloreactivity of T-cells lacking HDAC11 was detected in an in vivo model of acute GVHD.

HDAC11 was also shown to be involved in the functioning of Treg cells through the regulation of Foxp3 [119]. Foxp3 plays a crucial role in Treg development and functions by regulating the expression of genes that determine the phenotype and suppressive activity of Tregs. Not only does HDAC11 regulate Foxp3 expression, but it can also bind to Foxp3 and promote its deacetylation. Deletion of HDAC11 in Foxp3+ Tregs enhanced Foxp3 expression and promoted the expression of other genes important to the development and maintenance of the Treg lineage and their suppressive role. Interestingly, the deletion as well as the pharmacological inhibition of HDAC11 promoted Treg-dependent long-term allograft acceptance. Furthermore, silencing of HDAC11 in Hodgkin lymphoma cells induced expressions of OX40 ligand and pro-inflammatory cytokines, including TNF-α and IL-17, which could generate a favorable anti-tumor response with more effector and fewer regulatory T-cells [120].

## 3. The Action of HDAC Inhibitors in the Maturation and Activation of T-Cells

HDAC inhibitors (HDACi) are compounds that not only block the histone deacetylase activity of HDAC proteins but also affect the interactions of HDACs with other components of multiprotein complexes and the acetylation status and stability of HDAC proteins themselves [121]. As HDACs are important players in T-cells, the effect of HDAC inhibition on T-cell biology is strong, including the activation and functions of both conventional and regulatory T-cells. The strongest effect of HDACi was described on tumor cells, and they have been used as drugs in T-cell malignancies. However, nontumor immune cells are also affected and the anti-inflammatory effect of HDACi was described, especially through the modulation of T-cell activation and enhancement of Treg suppressive function [121].

HDACi were shown to affect Treg, APC, and T-cell interactions. In Tregs, HDACi increase the FOXP3 acetylation and stability as well as its function as a transcription factor that promotes genes associated with Treg function and their suppressive activity [92]. In the presence of HDACi, there is a decrease in proinflammatory cytokine release in APC cells which influences their T-cell stimulatory function [122]. In T-cells, HDACi affect the T-cell development, the maintenance of the naïve T-cell compartment, T-cell activation pathways, and T-cell immune responses [121]. HDACi promote the early activation of CD4+ cells by enhancing the production of Th1 and Th2 lineage cytokines and promoting cytokine release. They affect the proliferation of activated naïve T-cells by inhibiting the production of IL-2 and down-regulation of CD25 and CD154 [123]. In the early differentiation and polarization of Th0 cells, HDACi can influence cytokine gene transcription and the balance of cytokine production [121]. The balance and lineage commitment regulation can be disturbed, leading to incomplete Th1 and Th2 differentiation. In naïve CD4+ T-cells, HDACi were proven to induce antigen-specific anergy by upregulating p21 expression [124]. HDACi use can also promote the generation of iTregs from conventional T-cells, affect Teff cell development and function, and suppress T-cell activation as well as memory cell formation [121].

HDAC inhibitors can impair not only T-cell activation but also intratumoral T-cell effector functions [125]. Not only may they increase T-cell cytotoxicity, but they can also reduce the suppressive nature of the tumor microenvironment by impairing regulatory T-cells and enhancing T-cell migration to the tumor site [125]. As a result, treatment with HDAC inhibitors enhances the infiltration of tumors by T-cells, increases the recognition of tumors by T-cells, and promotes the susceptibility of tumor cells to T-cell-mediated killing [125].

## 4. Conclusions

HDACs are important regulators of T-cell development and function. Disruption of HDACs has been identified to be involved in hematological disorders. In peripheral T-cell lymphoma (PTCL), HDAC2 was detected to be overexpressed and considered to be a prognostic marker, particularly for patients with the PTCL-NOS subtype [126]. In cutaneous T-cell lymphoma, upregulation of HDAC1 and HDAC6 was detected as a result of excessive autocrine production of IL-15 driven by disruption of the Zeb1 transcription factor binding to the IL-15 promoter [64]. Consequently, HDAC1 upregulation increased the expression of oncomir miR-21, which probably contributes to the malignant transformation of normal T-cells. In adult T-cell leukemia/lymphoma (ATL), HDAC8 expression was driven by an oncogenic cascade of FRA-2/JUND and SOX4 transcription factor, and— together with other two activated genes, GCKR and NAP1—was proven to affect ATL cell growth [127]. Additionally, high expression of SIRT1 was detected in ATL patients, and inhibition of this deacetylase with sirtinol inhibited the proliferation of cells and induced apoptosis by activation of the caspase family and degradation of SIRT1 in the nucleus [128]. Additionally, histone deacetylase inhibitors turned out to be promising agents for various T-cell malignancies. Many of them have already been used in the therapy of cutaneous T-cell lymphoma or peripheral T-cell lymphoma. Moreover, many of them have been tested in clinical trials, either alone or together with other therapies [129]. The more we know about the role of HDACs in normal and malignant T-cells, the more effective and specific therapies can be developed for the treatment of T-cell malignancies.

## Data Availability

Not applicable.
